# Intraoperative hypotension during liver transplant surgery is associated with postoperative acute kidney injury: a historical cohort study

**DOI:** 10.1186/s12871-020-01228-y

**Published:** 2021-01-11

**Authors:** Alexandre Joosten, Valerio Lucidi, Brigitte Ickx, Luc Van Obbergh, Desislava Germanova, Antoine Berna, Brenton Alexander, Olivier Desebbe, Francois-Martin Carrier, Daniel Cherqui, Rene Adam, Jacques Duranteau, Bernd Saugel, Jean-Louis Vincent, Joseph Rinehart, Philippe Van der Linden

**Affiliations:** 1 Department of Anesthesiology, Erasme Hospital, Université Libre de Bruxelles, Brussels, Belgium; 2 Department of Anesthesiology and Intensive Care, Hôpitaux Universitaires Paris-Sud, Université Paris-Sud, Université Paris-Saclay, Paul Brousse Hospital, Assistance Publique Hôpitaux de Paris (APHP), 12 Avenue Paul Vaillant Couturier, 94800 Villejuif, France; 3 Department of Digestive Surgery, Unit of Hepatobiliary Surgery and Liver Transplantation, Erasme hospital, Cliniques Universitaires de Bruxelles, Université Libre de Bruxelles, Brussels, Belgium; 4 Department of Anesthesiology, University of California San Diego, La Jolla, CA USA; 5 Department of Anesthesiology and Perioperative Medicine, Sauvegarde Clinic, Ramsay Santé, Lyon, France; 6 Department of Anesthesiology, Centre hospitalier de l’Université de Montréal, Montréal, Québec Canada; 7 Department of Hepatobiliary Surgery, Paul Brousse Hospital, Villejuif, France; 8 Department of Anesthesiology, Center of Anesthesiology and Intensive Care Medicine, University Medical Center Hamburg-Eppendorf, Hamburg, Germany; 9 Outcomes Research Consortium, Cleveland, Ohio USA; 10 Department of Intensive Care, Erasme Hospital, Université Libre de Bruxelles, Brussels, Belgium; 11 Department of Anesthesiology and Perioperative Care, University of California Irvine, Irvine, California USA; 12 Department of Anesthesiology, Brugmann Hospital, Université Libre de Bruxelles, Bruxelles, Belgium

**Keywords:** Acute kidney disease, Renal failure, Chronic kidney disease, Hemodynamic, Postoperative complications, Transplant, Intraoperative

## Abstract

**Background:**

Acute kidney injury (AKI) occurs frequently after liver transplant surgery and is associated with significant morbidity and mortality. While the impact of intraoperative hypotension (IOH) on postoperative AKI has been well demonstrated in patients undergoing a wide variety of non-cardiac surgeries, it remains poorly studied in liver transplant surgery. We tested the hypothesis that IOH is associated with AKI following liver transplant surgery.

**Methods:**

This historical cohort study included all patients who underwent liver transplant surgery between 2014 and 2019 except those with a preoperative creatinine > 1.5 mg/dl and/or who had combined transplantation surgery. IOH was defined as any mean arterial pressure (MAP) < 65 mmHg and was classified according to the percentage of case time during which the MAP was < 65 mmHg into three groups, based on the interquartile range of the study cohort: *“short”* (Quartile 1, < 8.6% of case time), “*intermediate”* (Quartiles 2–3, 8.6–39.5%) and “*long”* (Quartile 4, > 39.5%) duration. AKI stages were classified according to a “modified” “Kidney Disease: Improving Global Outcomes” (KDIGO) criteria. Logistic regression modelling was conducted to assess the association between IOH and postoperative AKI. The model was run both as a univariate and with multiple perioperative covariates to test for robustness to confounders.

**Results:**

Of the 205 patients who met our inclusion criteria, 117 (57.1%) developed AKI. Fifty-two (25%), 102 (50%) and 51 (25%) patients had short, intermediate and long duration of IOH respectively. In multivariate analysis, IOH was independently associated with an increased risk of AKI (adjusted odds ratio [OR] 1.05; 95%CI 1.02–1.09; *P* < 0.001). Compared to “*short duration*” of IOH, “*intermediate duration”* was associated with a 10-fold increased risk of developing AKI (OR 9.7; 95%CI 4.1–22.7; *P* < 0.001). “*Long duration”* was associated with an even greater risk of AKI compared to “*short duration*” (OR 34.6; 95%CI 11.5-108.6; *P* < 0.001).

**Conclusions:**

Intraoperative hypotension is independently associated with the development of AKI after liver transplant surgery. The longer the MAP is < 65 mmHg, the higher the risk the patient will develop AKI in the immediate postoperative period, and the greater the likely severity. Anesthesiologists and surgeons must therefore make every effort to avoid IOH during surgery.

## Background

Acute kidney injury (AKI) is a common postoperative complication following liver transplantation and is associated with increased morbidity, mortality and development of chronic kidney disease [1–5]. One of the most common diagnostic criteria used to classify AKI is the “Kidney Disease: Improving Global Outcomes” (KDIGO) system, which is based on changes in serum creatinine and urine output [6]. However, as urine output is rarely documented accurately in the perioperative setting, increases in serum creatinine are frequently used independently to define postoperative AKI (“modified” KDIGO classification).

Multiple studies have identified patient and donor risk factors for AKI following liver transplant surgery including among others, female sex, obesity, diabetes, high model for end-stage liver disease (MELD) score, large amounts of blood loss, use of hydroxyethyl starch solution, perioperative blood glucose variability, cold and warm ischaemia times, donor age and graft sizes [7–12]. Intraoperative hypotension (IOH), most often defined as a mean arterial pressure (MAP) ≤ 65 mmHg, has been shown to be one of the most important factors associated with postoperative AKI [13]. Numerous large retrospective studies have shown that IOH is associated with postoperative AKI after various types of non-cardiac surgery, [14–19] but data on such an association in liver transplantation remain scarce [20].

We therefore conducted a historical cohort analysis to evaluate the association between IOH and the development of postoperative AKI in patients undergoing liver transplant surgery.

## Methods

This single centre historical cohort study was approved by the Institutional Review Board of Erasme hospital on December 14, 2018 under the reference P2018/555 with a waiver of informed consent because of the observational and retrospective nature of the study.

We identified all liver transplant patients from 2014 (when the anaesthetic data for our patients started to be computerised) to 2019 with our dedicated operating room softwares (TrackPro® and UltraGenda®, Belgium). We then retrospectively analysed the patients’ electronic medical records, which include a continuous intraoperative recording of vital signs (Innovian® Perioperative Care, Dräger, Lübeck, Germany). All patients who underwent a liver transplant between January 1, 2014 and December 30, 2019 were included except those. With a preoperative serum creatinine value > 1.5 mg/dL, and any patient who underwent a combined transplantation procedure (liver-kidney, liver-heart or liver-lung).

### Anaesthetic protocol

Intraoperative anaesthesia was standardised according to institutional guidelines. Patients arrived in the operating room and were placed under an infrared heating lamp. Several non-invasive monitors were then applied: a 5-lead electrocardiogram (ECG), non-invasive blood pressure, rectal temperature probe, and a frontal electroencephalogram using bispectral index (BIS) monitoring (Aspect Medical System Inc, Natick, MA, USA). A bladder catheter was inserted. Vascular access consisted of one or two large bore peripheral venous catheters, right femoral artery and vein catheters, and right jugular vein catheter. The left femoral and internal jugular veins were not cannulated in case veno-venous bypass was required. A Swan-Ganz catheter (Edwards Lifesciences, Irvine, CA, USA) was inserted and haemodynamic interventions were guided using continuous cardiac index, stroke volume index, mixed venous oxygen saturation, central venous pressure, and MAP. More specifically, the protocol used was to strictly maintain MAP > 65 mmHg and fluids were administered if stroke volume index (SVI) was < 30 ml/m^2^ and/or CCI < 2 L/min/m^2^. Fluid administration consisted of a baseline infusion of a balanced crystalloid (Plasmalyte®, Baxter, Belgium) and compensation for blood loss via 250 ml fluid boluses of either Plasmalyte®, 3% modified gelatin, or 4% albumin (depending on patient conditions and physician preference).

Rapid infusers, perfusion heaters, and a cell saver were ready for use prior to induction. In case of active haemorrhage, anaesthetists typically guided blood product administration using ROTEM monitoring. General anaesthesia was induced with propofol or etomidate. Antinociception was achieved with a remifentanil infusion and anaesthesia was maintained with sevoflurane or desflurane depending on physician preference. Rapid sequence intubation was performed if patients had not fasted appropriately or if they had abdominal ascites. Neuromuscular blockade was achieved in all patients and controlled with a train-of-four monitor (TOF scan, Idmed, France). The choice of muscular relaxant was left to the discretion of the anaesthetist.

### Surgical procedure

Almost all the liver transplantation were performed by recipient hepatectomy without venous-venous bypass, using the vena cava–sparing technique and piggy-back reconstruction. Liver reperfusion was performed through the portal vein first followed by subsequent arterial reperfusion. Biliary reconstruction was carried out with an end-to-end choledochectomy without a T-tube.

Our immunosuppressive regimen comprised primarily tacrolimus with mycophenolate mofetil and prednisone. Tacrolimus trough levels were maintained at 5–10 ng/mL. Steroids were discontinued approximately 3 months after liver transplant surgery.

### Measurements and study outcomes

During surgery, MAP was continuously monitored through the femoral arterial line and recorded automatically during surgery at 30 second intervals by our anaesthesia information management system (Innovian, Dräger NV, Wemmel, Belgium). We extracted the raw values: all values < 30 and > 150 mmHg were considered to be artifacts and deleted. For each patient, we calculated the mean MAP value during the procedure and the percentage of case time during which the patient was hypotensive, defined as a MAP < 65 mmHg. IOH was then categorised into 3 levels based on the interquartile range (IQR) values of the study cohort for the percentage of case time during which patients were hypotensive, according to the methodology of Thacker et al. [21]: “short duration” of IOH (in the lower 25th percentile), “intermediate duration” (between the 25th and the 75th percentile) and “long duration” (within in the upper 75th percentile).

The primary outcome was the development of stage 1–3 AKI, defined using serum creatinine-based KDIGO definitions without taking into account diuresis (“modified” KDIGO classifications) because urine output is rarely documented accurately in the perioperative setting. The three modified KDIGO stages are: (1) Mild injury: creatinine increase of at least 0.3 mg/dl within the first 48-hours or 1.5 to 1.9 times the baseline level during the first postoperative week; (2) Moderate injury: creatinine increase of 2.0 to 3.0 times the baseline; and (3) Severe injury: creatinine increase of greater than 3.0 times the baseline, creatinine level of at least 4 mg/dl, or dependency on renal replacement therapy.

### Statistical analysis

The normality of continuous data was assessed using a Kolmogorov-Smirnov test. Normally distributed variables were compared using a student’s t-test and are expressed as mean ± standard deviation (SD) and those not normally distributed were compared using a Mann-Whitney U-test and are expressed as median [25% − 75%] percentiles. Discrete data were expressed as a number and percentage and compared using a Chi square or a Fisher’s exact test when indicated.

We used logistic regression modelling to evaluate the association between IOH and the development of postoperative AKI. Univariate logistic models were used to test for association with AKI using the following independent variables: sex, age, ASA class, weight, body mass index (BMI), Child-Pugh score, baseline serum creatinine and haemoglobin, MELD laboratory score, duration of anaesthesia, duration of surgery, fluid volumes (crystalloid, colloid, packed red blood cells, cell saver), estimated blood loss, diuresis, total fluid output, net fluid balance, use of vasopressors, mean case time with central venous pressure > 8 mmHg, preoperative use of different medications (Table 1), patient comorbidities (Table 1), donor age, donor BMI, postoperative fluid balance, use of cardiopulmonary bypass, presence of portal ischaemia or arterial ischaemia and any episodes of MAP < 65 mmHg. Variables significantly associated in univariate testing were then included in a multivariate logistic regression to evaluate their association with AKI. Risks of developing AKI based on the model are presented as odds ratios [ORs] and their 95% confidence intervals. Statistical significance was determined at the 0.05 level. All analyses were conducted with Minitab (Paris, France) and R (www.r-project.org).

**Table 1:** Baseline characteristics

Variables	No AKI (*N* = 88)	AKI (*N* = 117)	*p*-value*
Age (years)	57 [48–64]	57 [51–62]	0.85
Male (%)	61 (69)	83 (71)	0.76
Weight (kg)	75 [61–83]	83 [70–94]	**0.019**
ASA score (II/III/IV/V)	4/55/28/1	1/56/57/3	0.99
**Comorbid conditions**			
➢ Myocardial injury (%)	5 (6)	5 (4)	0.61
➢ Arterial hypertension (%)	52 (59)	73 (62)	0.88
➢ Heart failure (%)	1 (1)	1 (1)	0.71
➢ Hyperlipidaemia (%)	8 (9)	28 (24)	0.055
➢ Diabetes mellitus (%)	26 (30)	32 (27)	0.16
➢ Atrial fibrillation (%)	8 (9)	11 (9)	0.75
➢ COPD (%)	5 (6)	4 (3)	0.61
➢ Peripheral arteritis (%	4 (5)	5 (4)	0.76
**Medication**			
➢ β blocker (%)	42 (47)	50 (43)	0.77
➢ ACEI (%)	6 (7)	8 (7)	0.87
➢ ARB (%)	2 (2)	1 (1)	0.99
➢ Diuretics (%)	34 (39)	64 (55)	**0.039**
➢ Statin (%)	9 (9)	13 (11)	0.75
Child-Pugh score	7 [5-12]	11 [7-13]	**0.022**
MELD score	12 [9-20]	19 [14-29]	**0.014**
Fulminant hepatitis (%)	4 (5)	8 (7)	0.81
Haemoglobin (g/dL)	12.1 [9.5–13.6]	10.4 [8.9–12.7]	**0.014**
Creatinine (mg/dL)	0.90 [0.70–1.19]	1.00 [0.70–1.32]	0.061
HBV (%)	14 (16)	14 (12)	0.86
HCV (%)	17 (20)	23 (20)	0.23
Donor age (y)	56 [46–66]	57 [45–68]	0.38
Donor BMI (kg/m^2^)	25 [23-28]	26 [24-28]	0.14

* Univariate analysis

Data are listed as “value (%)” and or median [25–75 percentiles]. *AKI* acute kidney injury; *ASA* American Society of Anesthesiology physical status; *COPD* Chronic obstructive pulmonary disease ; *ACEI* angiotensin-converting enzyme inhibitor; *ARB* angiotensin II receptor blockers; *MELD* model for end-stage liver disease; *HBV* Hepatitis B virus; *HCV* Hepatitis C virus

## Results

**Fig. 1:**
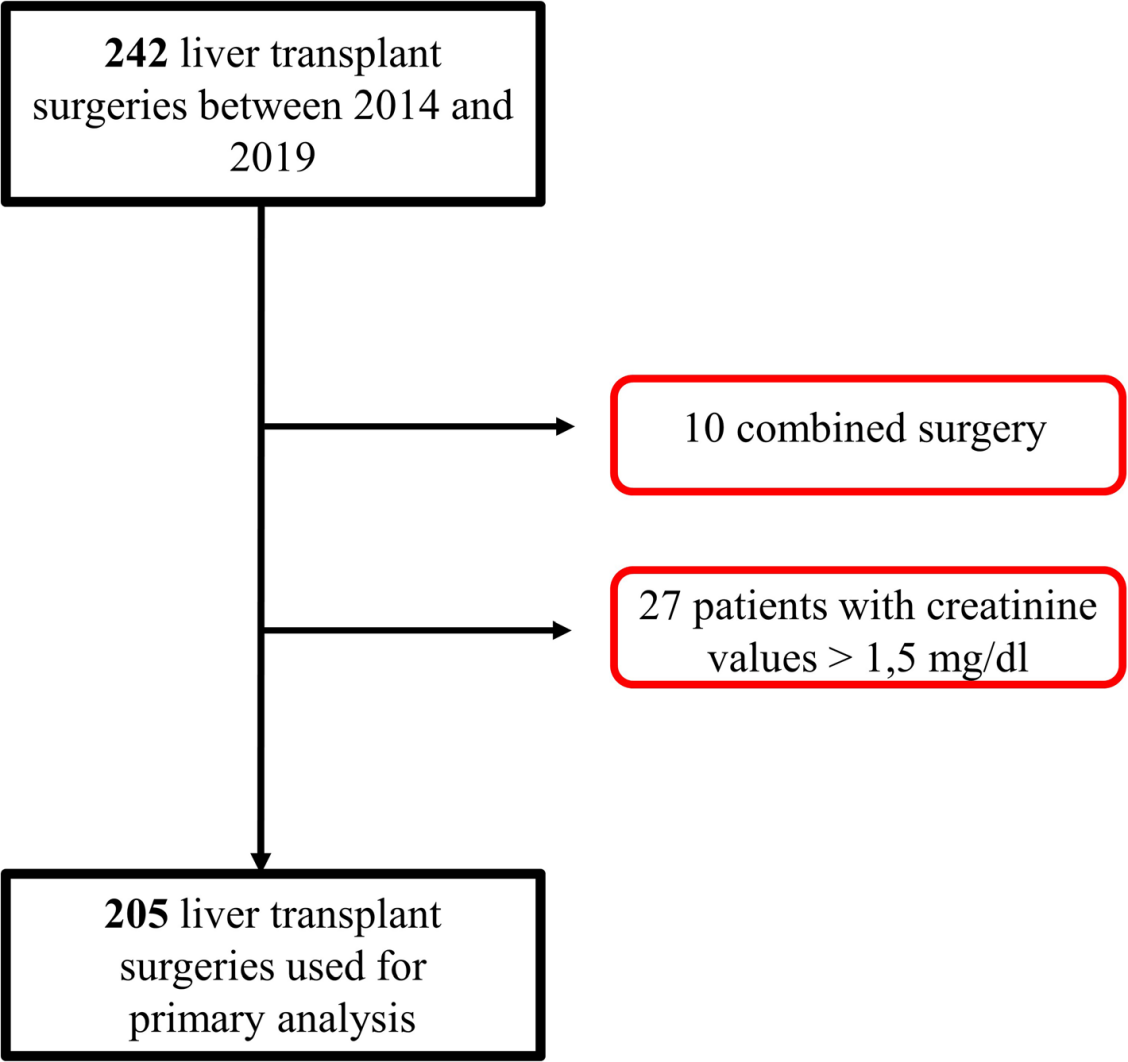
Flow chart

Among the 242 patients who underwent a liver transplantation between January 1st 2014 and December 30th 2019, 205 patients met our inclusion criteria (Fig. 1**)**.

One hundred and seventeen patients (57%) experienced some type of postoperative AKI (stages 1–3). AKI stage 1 occurred in 53 patients (25.9%) and stage 2–3 in 64 patients (31.1%). Among the whole study cohort, the median [25th − 75th quartiles] percentage of case time that patients had IOH was 21.4% [8.6–39.5]. Consequently, “short” duration of IOH was defined less than 8.6% of the intraoperative case time with a MAP < 65 mmHg (quartile 1), “intermediate” duration as 8.6–39.5% of case time with a MAP < 65 mmHg (quartiles 2–3) and “long” duration as > 39.5% of case time with a MAP < 65 mmHg (quartile 4). There were 52 (25%), 102 (50%) and 51 (25%) patients respectively each of these subgroups. Only two patients had no IOH using our definition (0% of case time spent with a MAP < 65 mmHg). Perioperative characteristics of the patients are shown in Tables 1 and 2.

**Table 2:** Perioperative variables

Variables	No AKI (*N* = 88)	AKI (*N* = 117)	*p*-value*
Mean MAP (mmHg)	78 ± 7	72 ± 6	**< 0.001**
Duration of IOH^a^ • Quartile 1 (< 8.6%) (%) • Quartiles 2–3 (8.6–39.5%) (%) • Quartile 4 (> 39.5%) (%)	44 (50) 37 (42) 7 (8)	8 (7) 65 (55) 44 (38)	**< 0.001**
Anaesthesia duration (min)	460 [411–536]	500 [453–583]	0.39
Surgical duration (min)	333 [294–385]	376 [324–438]	0.076
Venous bypass (%)	3 (3)	5 (4)	0.53
Portal ischaemia (min)	378 [319–458]	420 [360–495]	**0.047**
Arterial ischaemia (min)	28 [24-35]	33 [26-43]	0.25
Crystalloids (mL)	2500 [1500–4150]	2100 [1500–3774]	0.25
Colloids (mL)	650 [0-1075]	900 [300–1500]	0.30
Packed red blood cells (mL)	271 [0-1034]	753 [241–1339]	**0.013**
Cell saver (mL)	211 [0-710]	479 [0-956]	0.064
Total IN (mL)	6157 [4041–9726]	8068 [5285–11,517]	**0.015**
Estimated blood loss (mL)	2000 [1025–3000]	2700 [1500–5000]	**0.006**
Diuresis (mL)	368 [203–733]	259 [158–425]	0.26
Total OUT (mL)	2338 [1650–3304]	3050 [1793–5285]	**0.011**
Intraoperative net fluid balance (mL)	3660 [2028–5825]	4684 [2551–7764]	0.087
Fluid balance at POD#1 (mL)	1204 [188–2813]	2899 [1587–4534]	**0.002**
Combined fluid balance (mL)^b^	4926 [3103–8674]	8369 [4944–11,308]	**0.003**
Calculated blood loss (mL) at POD#2	777 [535–1450]	1222 [698–2080]	**0.010**
Mean central venous pressure > 8 mmHg^c^	50 (58)	75 (64)	0.336
Use of vasopressors	85 (97)	117 (100)	**0.044**

* univariate analysis

*IOH* intraoperative hypotension; *POD#1* postoperative day 1; *POD#2* postoperative day 2

^a^percentage of surgical time spent with a MAP < 65 mmHg (see text for details)

^b^combined fluid balance is the combination of intraoperative fluid balance and fluid balance on POD#1

^c^mean central venous pressure is the average of all values over the surgery

“Total IN” is the sum of crystalloid, colloid, packed red blood cells and cell saver administration and “total OUT” is the sum of estimated blood loss and urine output. Net fluid balance is the difference total IN – total OUT

Data are expressed as mean ± standard deviation, median and [25th -75th ] percentiles or number and percentage (%)

In univariate testing **(**Tables 1 and 2**)**, patients who developed postoperative AKI had higher BMI (*p* = 0.041), were more likely to have received preoperative diuretics (*p* = 0.039), had higher Child-Pugh (*p* = 0.0022) and MELD (*p* = 0.014) scores, had lower preoperative haemoglobin levels (*p* = 0.014), were more likely to have had prolonged IOH (*p* < 0.001), portal ischaemia (*p* = 0.047), or packed red blood cell transfusion (*p* = 0.013), and had higher total fluid input (*p* = 0.015), estimated blood loss (*p* = 0.006), and total fluid output (*p* = 0.011) than patients who did not develop AKI.

In multivariable analysis using the perioperative variables shown in Tables 1 and 2, only BMI and IOH (OR = 1.05 [1.02–1.09], *p* < 0.001), were significantly associated with an increased risk of AKI. For every one percent increase in case time spent with a MAP of ≤ 65 mmHg, the risk of AKI increased by about 5%.

**Fig. 2:**
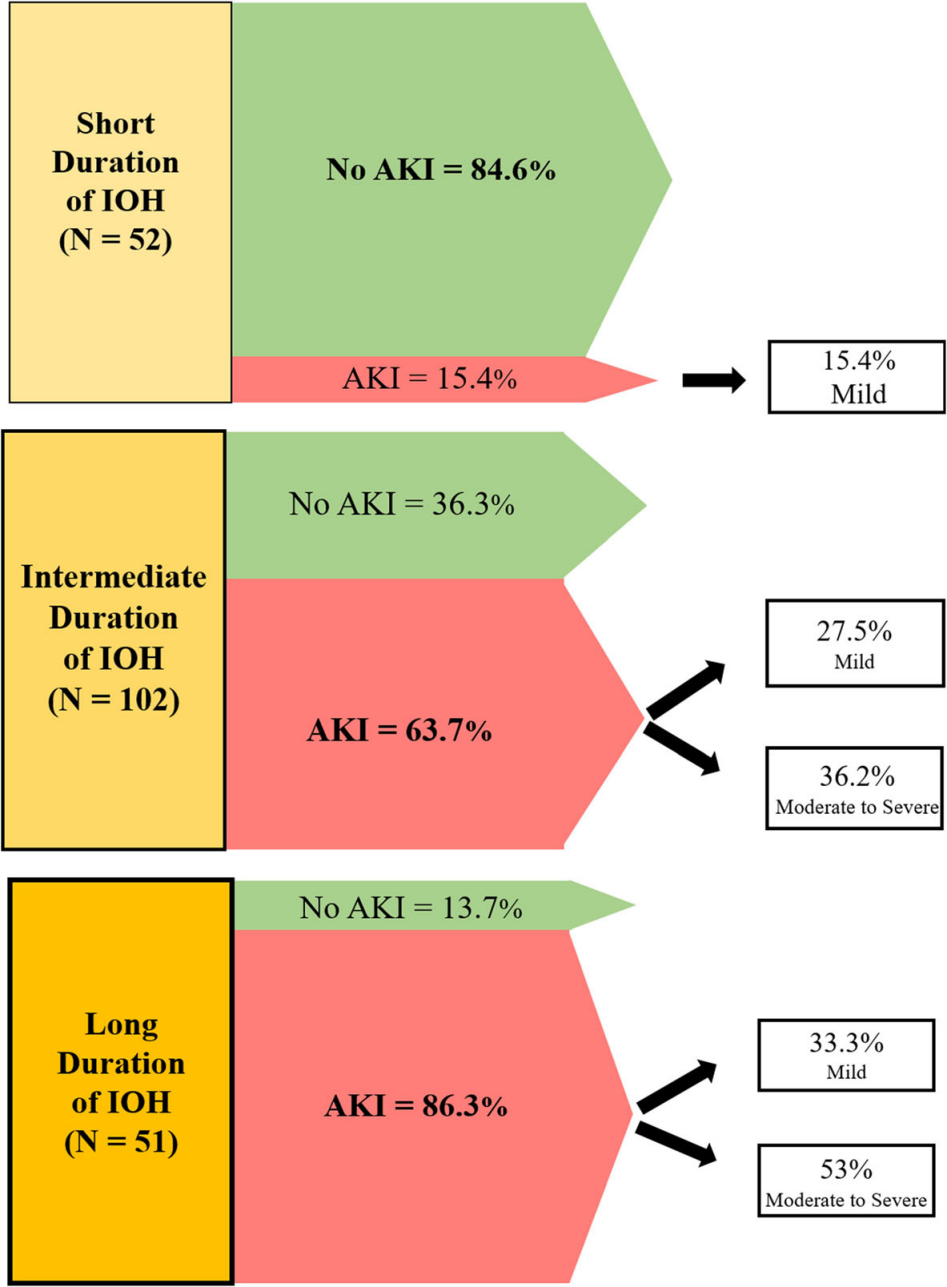
Duration of intraoperative hypotension and severity of acute kidney injury

Compared to “*short duration*” IOH, “*intermediate duration”* IOH was associated with a 10-fold increased risk of developing AKI (OR of 9.7; 95% CI 4.1–22.7; *P* < 0.0001). “*Long duration”* IOH was associated with an even greater risk of postoperative AKI (OR 34.6; 95% CI 11.5–108.6; *P* < 0.0001). Figure 2 shows the three different durations of IOH and their associations with the development of postoperative AKI. This suggests that the observed association between IOH and AKI was a length-response relationship. The longer a patient experienced a MAP < 65 mmHg during the liver transplantation procedure, the greater the risk of developing AKI in the immediate postoperative period. Patients who experienced a short duration of IOH (less than 8.6% of the surgery time) developed either no AKI (84.6%) or mild AKI (15.4%), while patients who experienced the largest duration of IOH (> 39.5% of the case time) developed AKI very frequently (86.3%). Among these patients who developed AKI, the majority experienced a moderate to severe AKI.

## Discussion

The presence of IOH was associated with an increased risk of developing postoperative AKI after liver transplantation and this association was independent of potential perioperative confounders. Moreover, the longer a patient spent with a MAP < 65 mmHg during the liver transplantation procedure, the greater the risk he or she had of developing AKI in the immediate postoperative period.

Several large retrospective studies have shown an association between IOH and postoperative AKI, [13–20, 22] and others have reported an association between the duration of IOH and cardiac, renal and neurological adverse events [13, 17, 23, 24]. A recent a systematic review including 42 studies has clearly confirmed the strong association between the severity and the duration of intraoperative hypotension and the increased risk of death or of developing end organ injury [23]. Indeed, the authors reported that is risk was already slightly increased when MAP was sustained at less than 70 mmHg for just 10 minutes. It was moderately increased with exposures to MAP less than 65 to 60 mmHg for at least 5 minutes, or any exposure to MAP less than 55 to 50 mmHg. High risk of any end-organ injury was reported for exposures to MAP less than 65 mmHg for at least 20 minutes, MAP less than 50 mmHg for at least 5 minutes, or any exposure to MAP less than 40 mmHg. Surprisingly, this association remains poorly defined in the context of liver transplantation [20]. To our knowledge, only one study has assessed the relationship between IOH and the risk of AKI in this patient population [20]. In that study, the authors demonstrated that severe IOH, defined as a MAP < 50 mmHg was strongly related to the development of moderate or severe AKI (stage 2–3). Our results are in line with this study and the others, and confirm that IOH is of significant clinical importance during liver transplantation, and that IOH should not be overlooked during the intraoperative period.

Patients undergoing liver transplant surgery frequently experience IOH as a result of various factors, including, among others, the duration of surgery, the severity of bleeding, the severity of the ischaemic reperfusion syndrome and the severity of the end-stage liver disease, characterized by a hyperdynamic state (high cardiac output and low systemic vascular resistance) [25–31]. However, most studies that have assessed predisposing factors for AKI after liver transplantation focused mainly on preoperative factors, which are often *not or poorly modifiable*. Intraoperative risk factors, such as IOH, are, in contrast, potentially modifiable, and may be minimised by close a collaboration between the surgeon and the anaesthetist. Our results suggest that avoiding or at least minimising the duration of IOH may be a valuable target to reduce the development of postoperative AKI.

Importantly, Erasme hospital has not developed strict protocols to avoid IOH during liver transplant surgery. Management of MAP is left to the discretion of the anaesthetist in charge of the patient, with the objective of avoiding a MAP < 65 mm Hg. The question of whether targeting a higher MAP (e.g., 80–85 mmHg) may decrease the incidence of postoperative AKI needs, however, to be considered. Indeed, two large randomised controlled trials have demonstrated that targeting a higher arterial pressure during surgery (well above 65 mmHg) was associated with a lower incidence of postoperative AKI [32, 33]. In the first, there was a lower incidence of organ dysfunction in the group of patients managed using a targeted systolic arterial pressure closer to the patient’s baseline value compared to the control group in which the same blood pressure target was used for all patients [32]. In the second study, targeting a MAP level between 80–95 mmHg in chronically hypertensive patients reduced the occurrence of postoperative AKI compared to two other MAP targets (65–79 and 96–110 mmHg) [33]. French national guidelines recommend maintaining of MAP > 70 mmHg in patients with chronic hypertension (which is the case in 60% of our study cohort) in order to prevent AKI [34]. It naturally follows that targeting a strict MAP goal of 65 mmHg can potentially be flawed as a strict definition of IOH is quite challenging. While some authors use a reduction from baseline value” (e.g. a 20–30% reduction from the patient’s preoperative MAP value), others continue to use the well-known “absolute” threshold value of 65 mmHg to define IOH. We decided in this study to choose the latter as this is the most common practice at our institution. The validity of this threshold can of course be challenged, but Salmasi and colleagues demonstrated that management based on an absolute MAP threshold of 65 mmHg in all patients was equivalent to management targeting relative reductions in MAP from preoperative values in terms of incidence of myocardial and kidney injury [13]. Additionally, although the results of a large randomised controlled study supported the individualization of arterial pressure targets in order to reduce the incidence of organ dysfunction (including a reduction in AKI), [32, 35] it is important to remember that such an approach can be extremely challenging to apply in patients undergoing liver transplant surgery, as higher values may potentially increase bleeding, making surgical conditions more challenging. As always, the risk-benefit ratio should be carefully assessed and future investigation into an optimal definition of IOH is urgently required for liver transplant recipients. Finally, the first author of this manuscript (AJ) has significant experience designing and using automated closed-loop systems to simplify and standardize the titration of hypnotics, fluid and vasopressors in the operating room [36–46]. We have recently demonstrated that such automated systems clearly outperform manual administration of hypnotic and vasoactive medications for maintaining a target variable (bispectral index, stroke volume index or MAP) within a narrow range [36, 38, 47]. In the future, this might be an appealing strategy to investigate the impact of such systems in this specific patient population who are at very high-risk of postoperative complications.

This study has several additional limitations that should be taken into consideration when interpreting our results. First, it was observational, historical, single-centre and included a relatively small sample size. Therefore, a causal relationship cannot be established and our results may not be generalisable to other hospitals with different perioperative haemodynamic and anaesthetic management. Second, our findings may be biased by unmeasured confounding parameters at both the patient and hospital levels. Third, as urine output was not taken into account for the classification of AKI, this may have led to a slight “underestimation” of the incidence of postoperative AKI in our study cohort, although a low urine output during the perioperative period is not always a marker of AKI. Fourth, per KDIGO definitions, we defined AKI as the change in creatinine value between the preoperative value and the highest value during the first postoperative week. This might introduce time-varying confounding or mediating factors, which limit interpretation of the study findings. Fifth, based on our study methodology, we voluntary excluded patients with pre-existing renal impairment. This could potentially explain why some well-known risk factors of postoperative AKI (e.g., MELD score, duration of surgery, duration of ischemia, age) did not emerge as significant in our analysis, although the number of excluded patients was quite small (27 patients). Sixth, postoperative hypotension was not taken into account as MAP was less frequently measured in the intensive care unit or on the floor than in the operating room. Seventh, although all patients had a pulmonary catheter, data on mixed venous oxygen saturation (SvO_2_), stroke volume index, continuous cardiac index and systemic vascular resistance were not collected by our electronic medical records (EMR) and thus, could not be assessed consistently in the present study. This is important as IOH should not always be treated with vasopressors and could be the result of hypovolemia or low cardiac output. It is interesting to note that a recent study demonstrated that decreased SvO_2_ was associated with postoperative AKI after liver transplantation [48]. Additionally, the total and maximum dose of catecholamines are also not reported here due to the same problem of a lack of connection between administration syringes and our EMR. Eighthly, we had no data on the occurrence of post- reperfusion syndrome and its importance on IOH duration. Finally, it is important to note that we reported the odds ratio for a frequent outcome (AKI), and the odds ratio can overestimate the risk in this situation.

## Conclusions

Our findings indicate that IOH is independently associated with the development of AKI after liver transplant surgery. The longer the MAP stays < 65 mmHg, the higher the risk the patient will develop AKI in the immediate postoperative period, and the greater the likely severity. Avoidance of IOH during liver transplant surgery may thus help reduce the incidence of this severe postoperative complication. A large prospective randomized controlled study with two different MAP levels (e.g., 60–65 mmHg vs. 80–85 mmHg) is needed to determine if targeting a higher MAP can reduce the risk of postoperative AKI.

## Data Availability

By request to the corresponding author.

## Abbreviations

IOH: Intraoperative hypotension
AKI: Acute kidney injury
KDIGO: Kidney Disease: Improving Global Outcomes
MELD: Model for end-stage liver disease
BMI: Body mass index
MAP: Mean arterial pressure
SVI: Stroke volume index
SvO2: Mixed venous oxygen saturation
EMR: Electronic medical record
